# Construction of Sui Generis Supervision System for Gene-Edited Animals in China

**DOI:** 10.3390/ani16050733

**Published:** 2026-02-26

**Authors:** Wenfei Zhang, Yaxin Song

**Affiliations:** 1 College of Humanities & Social Sciences, Huazhong Agricultural University, Wuhan 430070, China; songyaxin@webmail.hzau.edu.cn; 2 Agricultural and Rural Rule of Law Innovation Research Center, Huazhong Agricultural University, Wuhan 430070, China

**Keywords:** gene-edited animals, sui generis supervision system, eclectic supervision, categorization, animal welfare, full-process traceability, food labeling

## Abstract

As an important achievement in agricultural sci-tech innovation, gene-edited animals are not only a basic element for rural revitalization but also the core of promoting the biological breeding industry. Exploring their supervision models and institutional construction is practically significant and supports new productive forces in agriculture. Currently, various research and development achievements have emerged, but China lacks clear legal norms for their transformation and food industry supervision, failing to meet modern breeding needs. Internationally, three main supervision models (strict, open, eclectic) are determined by each country’s technological level, legal soundness and public acceptance. To build China’s sui generis supervision system for gene-edited animals that is independent of both GM animals and gene-edited plants, top-level design should issue categorized supervision policies to clarify research and development and industrialization norms, including relevant principles and procedures; establish a pre-positive ethical review mechanism with strict animal welfare checks; set animal welfare damage as a certification revocation condition; build a traceability platform for gene-edited animals and their products; implement positive food labeling; strengthen public participation to balance technological innovation and risk prevention; safeguard biosafety and public interests; and provide legal support for industrialization of gene-edited animals.

## Simple Summary

Gene-edited animals are obtained by modifying their own genes through gene-editing technology to acquire improved traits, and they have broad application prospects in both agricultural production and the biomedical field. Unlike genetically modified (GM) technology, gene editing carries lower technical risks and higher public acceptance, making the direct application of the GM supervision system inappropriate. Gene-edited animals are also distinct from gene-edited plants. Animals pose a risk of “escape” into natural ecosystems, and there are stricter ethical and social requirements such as laboratory animal welfare. Therefore, the supervision systems for gene-edited animals and plants should be differentiated. Grounded in the rapid development of emerging biological breeding technologies, this paper compares the supervision models for gene-edited animals in typical countries or regions. It proposes that China should gradually shift from a strict supervision model to an eclectic one and establish a sui generis supervision legal system for gene-edited animals under perspective from categorization, that is unique and independent of supervision systems for both GM animals and gene-edited plants. Meanwhile, it suggests strengthening the supervision of animal welfare and traceability labeling, so as to provide a critical path for balancing technological innovation, animal welfare and biosafety.

## 1. Introduction

### 1.1. Background

On 1 January 2025, the Central Committee of the Communist Party of China and the State Council issued *the Opinion on Further Deepening Rural Reform and Solidly Advancing the Comprehensive Revitalization of Rural Areas*, stating: “Promote collaborative research efforts in agricultural science and technology forces and continue to promote the industrialization of biological breeding” [1]. Modern biological breeding, abbreviated as biological breeding, primarily involves applying emerging biological breeding techniques such as genome-wide selection, synthetic biology, and gene editing to animals, plants, and microorganisms. This enables efficient, precise, and directed genetic improvement and breeding of varieties. Presently, the world has entered a new stage of multidisciplinary breeding characterized by the integrated development of “conventional breeding + biotechnology + information-based breeding”. Breeding platforms in China are also undergoing continuous refinement, with biological breeding techniques achieving sustained breakthroughs [2]. Since the 1980s, China has undertaken multiple major initiatives in relevant fields, including *the 863 Program*, *the 973 Program*, and *the Science and Technology Innovation 2030–Agricultural Bio-Breeding initiative*, with investment steadily increasing. These research projects provide vital financial and technical support for the development of China’s biological breeding industry. These initiatives have served as an “accelerator”, propelling substantial advancements in multiple aspects of biological breeding, including gene mining, genetic transformation, the breeding of new varieties, and safety assessments [3].

As a pivotal technology in biological breeding, gene editing has demonstrated immense potential for the genetic improvement of plants and animals, due to its high efficiency and precision. It has become an important foothold for scientific advancement across nations. However, China has not yet established a dedicated supervisory system for the experiments and industrialization process of gene-edited animals. Research units engaged in researching and developing gene-edited animals are currently conducting safety assessments and submitting applications under the framework for genetically modified (GM) animals, failing to reflect the particularity of supervision of gene-edited animals. In addition, the protection of intellectual property rights for gene-edited animal achievements is not strong enough, leading to a series of pain points such as ineffective breakthroughs in key technologies and insufficient collaboration among major bodies [4], which is not conducive to the acceleration of the industrialization process of biological breeding.

The United States (U.S.), Japan, and other countries have successively approved the marketing of various gene-edited plants and animals. For example, on 29 April 2025, the U.S. Food and Drug Administration (hereinafter referred to as the FDA), following its assessment, concluded that gene-edited pigs resistant to porcine reproductive and respiratory syndrome (PRRS) exhibited no abnormalities in growth, reproduction, or mortality rates, showing no significant differences from conventional pigs. Their muscle tissue composition and meat quality were indistinguishable from conventional pigs, posing no food safety risks to consumers or animals. Furthermore, the genotype and phenotype were found to be stably inherited across multiple generations of pigs. Consequently, the FDA approved these gene-edited pigs for breeding and food use [5].

Gene-edited animals offer novel opportunities in vital fields such as agricultural production and biomedicine. However, the unique supervision pain points of gene-edited animals impose new demands on emerging biotechnology supervision systems. Striking a balance between technological innovation, animal welfare protection, and risk prevention and regulation has become an urgent priority.

### 1.2. Literature Review

In the field of biological breeding, compared to traditional GM technology, gene-edited technology enables efficient, cost-effective and targeted editing of multiple genes without introducing exogenous genes. This approach facilitates more precise modifications to specific endogenous genes, thereby influencing and altering their molecular functions to achieve desired traits in organisms [6,7]. At present, the majority of scholarly research primarily focuses on the principles of gene editing [8], the developmental trajectory of gene editing technology itself [9,10], ethical risks [11,12,13], public acceptance and awareness [14], traceability mechanisms within the industrial chain and product labeling mechanism [15,16], the necessity for effective and practicable supervisory policies, and their critical significance for both fundamental innovation and practical application [17,18].

Gene editing technology is now increasingly applied in animal breeding, yielding remarkable research outcomes. For instance, Professor Gao Zexia’s team at Huazhong Agricultural University developed the “Wuchang fish without intermuscular spines” by knocking out the *runx2b* gene, thereby eliminating its intermuscular spines. This modification caused no significant impact on fish growth, the formation of other skeletal elements, or the fatty acids and amino acid content of its muscles [19]. For another example, a research team from Northwest A&F University has developed anti-mastitis gene-edited dairy goats with significantly enhanced resistance to mastitis. Through regulating sequence gene editing, they carried out targeted integration of the innate inflammatory regulatory sequences into the promoter region of the *lysozyme* gene. Moreover, the efficient expression of *lysozyme* genes under specific conditions was achieved by using the novel genome editing tool ISDra2-TnpB. Following *E. coli* infection, this approach effectively suppressed the activation of pan-apoptosis, mitigated damage to the haemolacteal barrier, and markedly reduced both the incidence and severity of mastitis [20].

With the advancement of gene editing technology and breakthroughs in animal innovation achievements [21], some scholars have also begun to focus on the particularities of supervision over gene-edited animals [22]. Furthermore, given the inherent safety risks of gene editing technology [23] and the greater ethical concerns associated with gene-edited animals compared to gene-edited plants [24], it is necessary to establish a specialized supervisory framework for gene-edited animals. This entails strengthening the top-level design of supervision for gene-edited animals, advancing supranational governance in this supervision domain, and setting prior ethical review procedures for animal welfare. While promoting their industrial application, it is essential to balance technological innovation with research ethics, thereby effectively guaranteeing national food security and biosafety.

### 1.3. Methods

#### 1.3.1. Literature Analysis

Through analysis of relevant domestic and international literature, the foundational theories were systematically reviewed and consolidated. The primary sources of literature were China National Knowledge Infrastructure (CNKI) and Multidisciplinary Digital Publishing Institute (MDPI). The search period spanned from 2010 to 2025, with keywords including “gene-edited animals”, “gene editing technology”, “animal welfare”, “gene-editing supervision”, etc. The literature selection criteria encompassed peer-reviewed academic journal articles, authoritative legal monographs, and official government documents. The analysis focuses on two specific dimensions: first, teasing the conceptual characteristics of gene-edited animal technology and its distinguishing features relative to GM technology, clarifying the independence and necessity of gene-edited animal supervision; second, systematically summarizing research findings on risk types of gene-edited animals, supervisory models in different countries, and public perceptions concerning gene-edited animals, thereby providing theoretical underpinnings and problem-oriented insights for establishing a categorized supervision system for gene-edited animals in China.

#### 1.3.2. Normative Analysis

Through the structured approach of “identifying problems, analyzing problems, solving problems” in law, this paper employs normative analysis to evaluate China’s supervision policies and existing legal norms governing gene-edited animals. Primary data sources include the China National Legal Database and official government websites such as the Ministry of Agriculture and Rural Affairs of the People’s Republic of China. The specific research encompasses: First, an evaluation of the predicaments encountered when applying existing Chinese laws and regulations to the supervision of gene-edited animals. This paper analyzes the legislative limitations arising from the failure to distinguish between GM technology and gene editing technology, alongside institutional gaps in animal ethics. Second, based on the current legislative loopholes and practical requirements, this paper undertakes an institutional analysis at the normative level to improve supervision legal system for gene-edited animals in China, focusing on core elements such as classification criteria for categorized supervision, prior ethical review procedures, full-process traceability, and food labeling mechanisms.

#### 1.3.3. Comparative Analysis

To draw on advanced extraterritorial experience and establish an international comparative perspective for China’s institutional construction, this paper employs comparative analysis to examine gene-edited animal supervisory models in typical countries and regions. Data on the current legal and supervision frameworks of various countries primarily originates from official websites such as the United States Department of Agriculture (USDA), the Ministry of Agriculture, Forestry and Fisheries of Japan (MAFF), and the European Food Safety Authority (EFSA). The selection of research subjects is based on the representativeness of their supervisory models (strict, open, or eclectic) and their level of technological development. The dimensions of comparative analysis focus on the positioning and core idea of each supervisory model, the primary laws and regulations, the design and implementation of safety assessment, animal welfare and food labeling. The study aims to reveal the underlying policy logic of different supervision models, which provides targeted comparative legal enlightenment for constructing a categorized supervision system for gene-edited animals in China.

## 2. Theoretical Justification of the Sui Generis Supervision of Gene-Edited Animals in China

### 2.1. Conceptual Distinction in Gene-Edited Animals

Gene-edited animals are essentially different from GM animals and gene-edited plants. For details, please refer to Table 1.

#### 2.1.1. The Particularity of Gene Editing Technology Compared with GM Technology

Gene editing technology refers to techniques that cut at specific genetic loci within organisms, causing double strand breaks in DNA, thereby through induced repair mechanisms, enabling directional transformation such as gene knockout, precise insertion, or replacement to obtain specific traits [34]. In recent years, with the optimization and iterative development of the CRISPR/Cas9 system and the continuous emergence of editing tools such as prime editing (PE), gene editing has rapidly progressed from fundamental research towards practical applications, demonstrating particularly significant potential in the field of animal breeding [35,36]. GM technology refers to the use of genetic engineering techniques to import and integrate target genes into the genome of a target organism, thereby modifying the original traits of laboratory animals or conferring new, superior characteristics, whilst ensuring these traits are stably inherited.

Traditional GM technology focuses on artificially introducing exogenous genes, i.e., transferring a gene controlling a particular trait from species A into species B. This enables species B to acquire that trait, ultimately achieving gene transcendence across species isolation. In contrast, gene editing technology primarily targets specific loci within the species’ own targeted genes to make edits. Exogenous DNA sequences function only during the editing process; once editing is complete, these sequences are separated and no longer exist, so they would not be inherited. The edited site, however, can be stably inherited. Due to this fundamental difference, the core points of contention in their supervision system differ, and the two should not be treated as equivalent.

This core distinction—whether exogenous DNA is stably integrated—results in fundamentally different end products. GM animals must contain detectable exogenous DNA. In contrast, many gene-edited animals only contain modifications to their endogenous genomes, with their products potentially indistinguishable from those of natural mutants. This essential difference determines the fundamental dividing line in supervisory logic between the two: supervision should focus on whether the final product contains stably inheritable exogenous DNA, rather than the broad sense of the “genetic manipulation” process itself, and the two should not be treated equally. Under the legislative model and supervision system for gene-edited biosafety exemplified by the U.S., CRISPR-Cas9 gene-edited organisms that contain no “newly introduced genetic material” or exogenous DNA are significantly different from GMOs. Consequently, they may be exempt from regulation by the Animal and Plant Health Inspection Service [37].

#### 2.1.2. The Particularities of Gene-Edited Animals Compared with Gene-Edited Plants

Gene-edited animals refer to those in which one or several genes with specific functions in an animal are targeted and modified by gene editing methods, ultimately influencing the physiological traits of animals. Genetic variation serves as the basic resource for animal trait improvement and molecular breeding. The exploitation of emerging breeding technologies is crucial for assisting breeding and genetic improvement. Currently, CRISPR-Cas9 System-based gene editing technologies in the animal sector are primarily applied to decode gene functions and enhance genetic breeding capabilities in livestock and poultry [38]. Gene-edited plants refer to the purposeful modification of plant genetic sequences using precise gene editing tools to improve plant varieties or achieve other specific objectives. In contrast to the nascent development of gene-edited animals, gene editing technology has become a powerful tool for breeding disease-resistant crops. It is now widely applied to major food crops such as rice, wheat, maize, and potatoes. Gene editing achieves site-directed modification of target genes through precise site-specific insertion, knockout, or modification, thereby optimizing agronomic traits in crops. This includes enhancing disease resistance, improving quality, and increasing yield [39].

A significant distinction between gene-edited animals and gene-edited plants lies in the potentially higher ethical risks associated with the former, namely, that gene-edited animals carry a strong ethical dimension, primarily focused on impacts on animal welfare and species ecosystems. The strong ethical nature stems from the fact that animals are perceptible subjects, involving pain perception, behavioral expression and psychological state. This requires that supervision must be established throughout the entire life cycle of animals, covering all aspects of experimental treatment and industrial processes, with a complexity far beyond that of plants.

First, animal welfare encompasses both material and spiritual dimensions, requiring the kind treatment of gene-edited animals. Specifically, the keeping and use of gene-edited animals must meet the following conditions: providing a clean and comfortable environment; supplying food, water, and sufficient space to maintain health; and minimizing unnecessary distress, discomfort, injury, disease, and pain [40].

Second, species ecosystems primarily refer to the uncontrollable impacts arising from the “escape” of gene-edited animals. This denotes the adverse consequences—such as threats to human safety, harm to ecological environments, and impacts on the scientific research—resulting from gene-edited animals, originally used for scientific research and other purposes, escaping artificial control due to accidental events. For instance, fish are extremely prone to “escape” during the breeding process as water sources flow and enter wild water bodies. And considering the domestication and selection of farmed species, “the escape” often has the advantages of fast growth and large body size. This enables them to secure more reproductive opportunities within wild populations, leading to unforeseeable impacts on subsequent population reproduction. This may cause population decline and generate external effects on the original ecosystem [41].

The strong ethical dimension also poses a major challenge to industrializing gene-edited animals and establishing a corresponding supervision system. Compounded by issues of food safety and the public’s right to informed consent, the supervision of gene-edited animals demands higher ethical scrutiny. So directly applying the supervision system for gene-edited plants to gene-edited animals would fail to provide appropriate oversight and achieve the desired outcomes.

### 2.2. The Types of Gene-Edited Animals and the Differences in Animal Welfare

According to different classification criteria, gene-edited animals can be divided into different species. The welfare and ethical considerations for gene-edited animals vary across species.

#### 2.2.1. Types of Gene Editing Technologies and the Differences in Animal Welfare

According to the targeted gene’s heritability, gene editing technology can be divided into hereditary gene editing and non-hereditary gene editing. The boundary of this classification depends on whether the gene-edited result can be transmitted to the next generation through natural reproduction. This classification of gene editing is mainly applied in diseases treatment. To date, more than ten gene replacement therapy and gene editing therapy products have been approved for marketing worldwide, mainly for the treatment of genetic diseases. With the development of gene editing technology, the application scope of gene therapy is gradually expanding from genetic diseases to common diseases [42]. For instance, Shanghai Tech University has delivered neurotrophic factors derived from nerve cells into the sheath of amyotrophic lateral sclerosis mice through AAV to treat their diseases [43], etc.

The extent to which hereditary gene editing and non-hereditary gene editing affect animal ethics and welfare is completely different. Non-hereditary gene editing takes animal somatic cells as the object and only affects the current generation of experimental subjects. The modified genes will not be passed on to the next generation through natural reproduction, causing less interference to the species’ gene pool. It only needs to focus on welfare issues at the individual level. For instance, physiological damage during the gene editing process, perception by experimental subjects, and postoperative quality of life. Hereditary gene editing usually targets germ cells or embryos. Genetic alterations will spread throughout the entire species population during reproduction, altering the original physiological characteristics and behavioral patterns of the species and affecting the survival competitiveness of the species population. The welfare ethical risks and ecological risks associated with this are significantly higher than those of the former.

#### 2.2.2. Types of Application Scenarios for Gene Editing and the Differences in Animal Welfare

The application of gene editing technology and its impact on animal welfare vary significantly across different application scenarios: laboratory, agricultural, and wild animals.

First, laboratory animals refer to those that have been artificially bred or modified for use in scientific research, teaching, production, verification, and other scientific experiments. Gene editing of laboratory animals is mostly confined to the laboratory environment, and these animals rarely enter the natural ecosystem. The scope of influence is relatively controllable, and the ethical risks are relatively small. In addition, due to the essential purpose of scientific research and experiment, the life cycle of laboratory animals faces a series of abnormal deaths such as euthanasia, which, to some extent, reflects the impairment of animal welfare. Therefore, the review of welfare ethics should focus on the animal welfare guarantee in the experimental process.

Second, agricultural animals refer to those artificially bred and raised to provide agricultural products and assist in agricultural production, also known as farm working animals. Gene editing of agricultural animals mainly serves production needs. Although it does not directly enter the population ecosystem, it must also consider the dual ethical dimensions of individual health and human food safety. With the development of modern animal husbandry, the intensive breeding model has increased the risk of infectious diseases transmission among livestock and poultry, seriously affecting animal breeding and human health. Gene editing technology offers new strategies and methods for disease-resistant breeding of livestock and poultry [44], but it also faces many challenges. For instance, gene off-targeting leads to alterations in gene function and the onset of diseases, causing issues such as animal welfare ethics.

Third, wild animals are defined as animals that grow in their natural state, are undomesticated, and live in natural environment. From the perspective of animal welfare, gene-edited wild animals should be encouraged to return to their natural habitats and maintain their natural ecological status so as to protect their rights to natural survival. However, when gene-edited individuals return to their original animal populations, the modified genes may be integrated into the wild animal gene pool, disrupting the original ecological balance. The ethical risks and potential hazards are even more prominent. In addition, the legal nature of gene-edited wild animals is still unclear, and the wildlife authorities have no authority to restrict gene-editing behavior [45]. Therefore, the application of gene editing technology in wild animals still poses significant unknown public health risks.

### 2.3. The Necessity and Feasibility of Sui Generis Supervision for Gene-Edited Animals

There are strong necessity and ample feasibility for the sui generis supervision of gene-edited animals. The term “sui generis” is independent of supervision system for GM animals and gene-edited plants, refers to a unique and special supervision system tailored exclusively for gene-edited animals. Such supervision will not give rise to systemic inconsistencies in the existing legal framework. This article paper adopts the term “supervision” (except where official expressions such as laws and regulations use other terms), which covers both oversight and management. It has a broad scope and emphasizes the diversity of regulatory subjects, not only limited to the management of government departments but also including the oversight of the public and others. “Management” and “regulation” usually specifically refer to the management responsibilities of administrative entities, such as government departments or companies. “Governance” generally refers to the way of governance or governance mechanism, weakening the function of supervision.

#### 2.3.1. The Necessity of Sui Generis Supervision for Gene-Edited Animals

Douglass North proposes that the demand for new institutions or rules usually arises when existing institutions and social rules are no longer adequate for the public to survive and prosper economically [46]. The institutional innovation of the sui generis supervision system for gene-edited animals is precisely because the existing institutional supply is unable to meet the real needs of technological development, as well as social and ethical demands.

First, gene editing technologies and gene-edited animals entail inherent risks, which creates a practical demand for supervision.

(1) Gene editing technology itself carries off-target risks. The CRISPR/Cas9 system is currently the most widely used method in the field of genome editing, enabling efficient gene editing and providing technical support for related research. However, this technology still exhibits “off-target effect”. The specificity of the CRISPR/Cas9 gene editing system is determined by the recognition sequence on the sgRNA. Within complex biological genomes, insufficient specificity of the sgRNA can lead to local matches between the recognition sequence and non-target DNA. Precisely because sgRNA can tolerate certain mismatches with the DNA target, this results in unintended off-target mutations, termed the “off-target effect”. This introduces significant uncertainty into gene editing outcomes [47].
(2) Gene-edited animals carry risks of alienation. Due to the complexity of intrinsic genetic mechanisms, the potential risks that poses by genetic modification to the organism itself remain unclear [48]. Compared to plants, gene editing in animals poses challenges such as embryo manipulation and the use of surrogate mothers, making the process more complex, costly, and time-consuming. Moreover, given the irreversibility of gene editing outcomes, current technological monitoring mechanisms may be insufficient to prevent potentially severe and unpredictable impacts on other species. This is because the application of gene editing technology will inevitably shift from repairing mutant genes to enhancing the original genes. The diffusion rate of such a process of gene introduction across the entire biosphere, along with its consequential effects on the whole biosphere, is difficult to assess and control. Deviation in gene editing carries a high risk of generating the alienation effect of technological application, including the evolution of superweeds, the increase in resistant pest populations, monoculture, biodiversity loss, disruption of food chains, and potential gene flow [49].

Second, gene-edited animals present substantial social and ethical risks, and society has called for appropriate supervision. Ethical concerns have long been a key issue and governance challenge in the development of emerging biotechnologies [50]. The internationally recognized “3R” principles [51] (“Replacement, Reduction, and Refinement”) regarding animal welfare and the five freedoms principles [52] (“freedom from hunger and thirst, freedom from discomfort, freedom from pain, injury, and disease, freedom to express normal behavior and the freedom from fear and pain”) provide a scientific ethical scale and operational guidelines for assessing the impact of gene editing technology on animal welfare. Gene-edited animals should give sufficient consideration to animal welfare, follow relevant principles, and design and complete experiments based on this.

However, the academic community currently pays more attention to the ethical issues raised by human gene editing, while those arising from gene-edited animals receive less attention. On the one hand, the research, development, and industrialization of gene-edited animals are closely related to human welfare. Gene editing in animals may induce permanent, irreversible alterations to the direction of animal evolution, thereby raising multifaceted issues including food safety, public informed consent, economic benefit distribution, environmental protection, and species ecological balance. On the other hand, gene-edited animals are related to animal welfare and animal rights issues. These encompass disease immunity and veterinary treatment protocols, humane treatment, and slaughter practices [53], as well as the physiological, psychological, and quality-of-life impacts of genetic editing on the animals themselves.

The social and ethical risks of gene-edited animals require a dialectical assessment from both short-term and long-term perspectives. This is particularly important given the complex duality inherent in animal welfare and animal rights. In the short term, gene-edited animals are expected to enhance animal welfare significantly. For instance, by equipping animals with innate resistance to specific diseases (such as PRRS and bovine mastitis) through gene editing, we can reduce the suffering caused by illness and improve the health and quality of life of individual animals. From a long-term perspective, however, potential risks and ethical uncertainties cannot be ignored. First, the uncertainty inherent in gene editing technology may lead to unexpected health problems in animals. Second, the potential impact of gene editing technology on ecosystems and natural species is unpredictable. If gene-edited animals accidentally escape or are approved for release into their original ecological environment, the modified genes may enter the wild populations through reproduction and affect the genetic structure of the native population.

China should enact specialized legislation for gene-edited animals, introduce targeted and forward-looking supervision policies, and strengthen legal supply to equalize supply and demand in the system, ensure the healthy and orderly development of gene-edited animals and their industrial applications [54].

#### 2.3.2. The Feasibility of Sui Generis Supervision for Gene-Edited Animals

First, there are institutional experiences worth learning for supervising gene-edited animals, and supervisory efforts in the field of gene-edited plants have yielded significant results. With the development of gene editing technology, numerous countries have revised legislation or issued new regulations specifically for gene editing technology to accelerate the commercialization of gene-edited crops. The U.S. regulates products based on the “substantial equivalence principle”; if a gene-edited plant is deemed “substantially equivalent” to one produced through conventional breeding methods, it is exempt from GMOs legislation [55]. China has strengthened institutional safeguards for biotechnology applications by adjusting a series of laws, regulations, and technical specifications. This approach draws upon the policies and regulations of typical nations and regions such as the U.S. and the European Union (EU), while adapting them to the current national circumstances of China.

Currently, safety assessments for agricultural gene-edited plants in China primarily focus on four aspects: molecular characteristics, genetic stability, environmental safety, and food safety. These are categorized based on whether exogenous genes have been introduced. Gene-edited plants introducing exogenous genes still undergo safety assessment applications in accordance with the *Guidelines for the Safety Assessment of GM Plants*. Conversely, gene-edited crops without introduced exogenous genes are exempt from such declarations required for GM crops. In January 2022, the Ministry of Agriculture and Rural Affairs of China formulated the *Guidelines for the Safety Assessment of Gene-Edited Plants for Agricultural Use (Trial)*, which has further shortened the approval cycle, accelerating the commercialization of gene-edited plants for agricultural use [56]. Meanwhile, for the first time in the plant sector, it formally distinguished the concept of “gene editing” from “GM” at the supervisory level. This provides an institutional foundation for the specialized oversight of gene-edited plants and offers a reference for the supervision of gene-edited animals. According to institutional migration theory, the effect of the supervision system for gene-edited plants can provide a feasible pathway for establishing a supervision system for gene-edited animals, thereby achieving overall consistency within the bio-safety legal system.

Second, there is scope for establishing a supervision system for gene-edited animals, which can be effectively aligned with the existing biotechnology supervision system. Sui generis supervision of gene-edited animals would not conflict with the existing legal system governing GMOs or gene-edited plants and could be accommodated within the current biotechnology supervisory system. Documents applied to gene-edited plants issued in China could provide directional and methodological guidance on supervising gene-edited animals. Examples include the idea of categorizing supervision and the essential elements of the safety assessment framework. Concurrently, China is actively advancing the formulation and publication of the *Guidelines for the Safety Assessment of Gene-Edited Animals for Agricultural Use*, aiming to conduct more professional and specific evaluations for gene-edited animals [57].

Moreover, relevant provisions concerning the ethical review of animals are stipulated in Chinese documents such as the *Regulations on the Management of Laboratory Animals* [58], the *Guidance on the Humane Treatment of Laboratory Animals* [59], the *Biosafety Law of the People’s Republic of China* [60], and the *Opinions on Strengthening the Governance of Scientific Ethics* [61]. Ethical review of animals constitutes an assessment and oversight of laboratory animals at the ethical level, compelling researchers to employ the best experimental methods, the optimal procedures, and the least-harmful means to achieve the best possible outcomes [62]. The review mainly focuses on the necessity of the experiment, compliance with the “3R” principles, and potential risks associated with the experiment. This ethical review system is widely accepted as the optimal approach to maximize high-quality science [63], thereby providing a possibility of connection for establishing a supervision system for gene-edited animals.

In summary, owing to technological uncertainties, ethical particularities, and insufficient supply in the current system, there is an urgent need to establish a sui generis supervision regime for gene-edited animals, which is an essential choice and institutional safeguard for ensuring the healthy development and safe, controllable application of gene-edited animal technologies. Such a system can also be integrated with existing biotechnology supervision and ethical review systems without causing institutional conflicts, thereby advancing technological development across relevant fields.

## 3. International Comparison and Chinese Choice of Supervision System for Gene-Edited Animals

Based on the varying degrees of strictness in supervising gene-edited animals across typical extraterritorial countries or regions, supervision models can broadly be categorized into three models: the strict supervision model, the open supervision model, and the eclectic supervision model.

### 3.1. Strict Supervision Model

This model typically treats gene-edited animals as equivalent to GM animals, imposing strict supervision throughout the entire process from research and development to industrialization, with greater emphasis on risk prevention. Given the comprehensiveness of its legal framework, this paper takes the EU as a representative example. It adheres to a relatively high level of risk control in the supervision of genetic technology and its products and has strict supervision of marketing approvals and labeling requirements.

The EU explicitly classifies gene-edited animals as GMOs subject to strict supervision, excluding them from the scope of novel foods. Novel foods are defined as “foods without a history of remarkable consumption within the EU”. Although novel foods also include the category “produced by new technologies” and both novel foods and GMOs require safety assessments, novel foods can benefit from relatively flexible supervision and a more streamlined approval process. The decision to separate gene-edited animals from novel food stems of the EU is to ensure their potential risks receive the highest level of control. Furthermore, the 2018 ruling by the Court of Justice of the EU reaffirmed that organisms obtained through mutagenesis techniques are considered GMOs and must comply with the *GMOs Directive* (*Directive 2001/18/EC*).

The strict supervision model of EU is applied throughout both the early research and development phase and the subsequent industrialization stage.

(1) In the early research and development stage, the EU has consistently attached great importance to animal welfare. The attainment of animal welfare has been incorporated into its supervision scope through the enactment of the new directive, the *European Community Regulation on Animal Protection Used for Scientific Purposes* (*2010/63/EU*) (hereinafter referred to as the *Animal Protection Regulation*). This mandates that all experiments involving animals must undergo ethical review and adhere to the “3R” principle and the Five Freedoms principles.

The *Animal Protection Regulation* further stipulates: “Member States shall ensure that each breeder, supplier and user sets up an animal-welfare body”, thereby guaranteeing the extensiveness and universality of animal welfare institutions. It further stipulates that animal welfare institutions should supervise the laboratory personnel, provide guidance, and submit records to the relevant authorities. Furthermore, the EU sets a “legal license” for research and development activities through project authorization and continuous supervision. This requires all animal experimentation projects to obtain a favorable project assessment from the authority responsible before implementation and to be conducted strictly in accordance with the authorization. Additionally, a “retrospective assessment” must be conducted upon project completion to ensure that the implementation of the project conforms to the values of animal welfare protection [51].

(2) In the later industrialization stage, the EU stipulates that food derived from gene-edited animals shall be subject to supervision under the laws governing GM food. Its strict supervision approach is reflected mainly in two aspects.

First, the approval for listing has become stricter. The supervision basis for gene-edited animal foods entering the market is *Regulation (EC) No 1829/2003* on the supervision of GM biological foods. This legislation mandates that gene-edited animal foods require authorization for market entry. Authorization applications are assessed by the European Food Safety Authority (EFSA), with the final decision on authorization resting with the European Commission. Beyond market authorization, pursuant to *EU Directive 2001/18/EC*, gene-edited animal foods must also undergo an environmental risk assessment before market entry. This assessment is conducted by the responsible authority of the Member State where the product first enters the market.

Second, stricter labeling requirements. Under *EU Regulations (EC) No 1829/2003* and *(EC) No 1830/2003*, gene-edited animal foods must be labeled to indicate they contain gene-edited animal tissue, or specify which ingredients are derived from gene-edited animals. However, labeling is not required if the presence of gene-edited animal tissue in food is incidental or technically unavoidable, provided the tissue content does not exceed 0.9% of the food or any component thereof. The EFSA supervision guidance on GM techniques also applies to the supervision of gene-edited animals, including the listing and environmental assessment of gene-edited animal food, etc. This guidance assesses gene-edited animal food relative to ordinary food and stipulates assessment stages, materials, and criteria. Under this strict supervision system, no gene-edited animal food has yet been approved for listing in the EU.

### 3.2. Open Supervision Model

This model is mainly based on the principle of substantial equivalence. If gene-edited animals are as safe as conventionally bred animals, or if the function of gene editing technology is essentially identical to that of conventionally breeding, the supervision of gene-edited animals will be streamlined, and measures such as exemption from labeling will apply. Greater emphasis is placed on encouraging technology transfer and product commercialization. This paper takes the U.S. and Japan as representative examples: the U.S. boasts relatively advanced gene editing technology is relatively high, while Japan has liberalized and streamlined its supervisory procedures to the greatest extent.

First, the U.S. adopts the “substantial equivalence principle” for supervising gene-edited animals. This means that during the review process for gene-edited animals, if products edited through genetic engineering achieve the same modifying effect as those produced via ordinary breeding, the gene-edited animals will not be subject to further regulation. In short, gene-edited animal products are subject to ordinary food supervision laws rather than GMOs supervision frameworks. Under the *National Bioengineered Food Disclosure Standard* of the USDA, the U.S. implements a “labeling exemption” policy for gene-edited foods. This means the public cannot learn through labeling that food is a gene-edited product, effectively depriving them of their right to know.

Regarding supervision bodies, the FDA and USDA have signed a Memorandum of Understanding to provide the industry with detailed information on the supervision of Heritable International Genomic Alterations in Animals (IGA). This clarifies the respective responsibilities of the FDA and USDA in supervising IGA and promotes information sharing and cooperation between the two agencies. In practice, the U.S. joined 12 other nations in 2018 to issue a statement opposing the unjustified differentiation of gene-edited products. In 2020, Galsafe pigs of Revivicor Company received approval for listing. By 2025, gene-edited pigs of Genus Company resistant to porcine reproductive and respiratory syndrome (PRRS) gained approval for listing, with anticipated arrival in the US market as early as 2026 [5]. This progression signifies that under a lightweight supervision policy, the approval for listing of gene-edited animals and their foods in the U.S. will increasingly become the norm.

Regarding the welfare of laboratory animals and ethical review, the U.S. enacted the *Animal Welfare Act* in 1966, mandating that all research institutions establish an effective Institutional Animal Care and Use Committee (IACUC). The Department of Agriculture further has 96 supervisors responsible for annual surprise inspections, with authority to impose fines for violations of this Act. Additionally, the Public Health Service Policy regarding the Humane Care and Use of Animals in Health Research, enacted under the *Health Research Act Supplementary Act* of 1985, similarly mandates that all research institutions establish animal care and use committees and implement the *Guidelines for the Care and Use of Laboratory Animals*. Furthermore, the non-governmental, non-profit organization Assessment and Accreditation of Laboratory Animal Care International participates in evaluating animal welfare conditions at over 600 research institutions and conducts inspections with appointment [64].

Second, the supervision of gene-edited animals employs a lenient system in Japan. Its safety assessment system for gene editing technology operates entirely independently of the safety assessment system for GMOs. Under Japanese legislation, such as the *Food Sanitation Act*, producers or developers seeking to market gene-edited animal products must first submit a pre-market consultation application to the Ministry of Health, Labor and Welfare. If assessed as containing no exogenous genes, gene-edited animal foods may enter the market by notifying the Ministry of Health, Labor and Welfare. The *Guidelines for the Safe Handling of Gene-Edited Foods and Food Additives* further stipulate that gene-edited organisms that do not introduce exogenous genes are not classified as GMOs. They are deemed to be as safe as foods developed using traditional breeding techniques, thus exempting them from the safety assessments required for GM foods. They may be sold upon just filing for the record. Under these provisions, developers of gene-edited foods may apply to solicit opinions from the government department during the research and development phase. The government will then refer such applications to the GM Food Investigation Committee, which will decide whether it is necessary to consult the Food Safety Committee [65].

Regarding food labeling, Japan’s legal provisions are largely consistent with those of the U.S. Under the *Food Labeling Act* and relevant documents from the Consumer Affairs Agency, gene-edited animal foods that do not contain exogenous genes may voluntarily indicate the presence of gene-edited animal tissue, with no mandatory requirements imposed. In practice, Japan approved the listing release of gene-edited tiger dolphins and red snapper possessing rapid growth traits in 2021. In 2023, it authorized the market release of gene-edited flatfish exhibiting accelerated growth. In 2025, new approval was granted for the market release of gene-edited tilapia with improved feed utilization efficiency and growth rates. Japan’s open supervision model has positioned it as the nation with the most gene-edited animal foods approved for listing globally. However, this development also highlights the practical challenges facing the aquaculture industry in Japan, including supply-demand imbalances.

### 3.3. Eclectic Supervision Model

This model mainly classifies products based on whether they contain exogenous DNA and oversees them through a “certification system” or an “exemption system”, among other measures. At the same time, it explicitly mandates the labeling of products derived from gene-edited animals, balancing risk prevention and control with innovation incentives, thereby reflecting a balance between safety and development. This paper takes post-Brexit United Kingdom, Australia and Thailand as representative examples, mainly on account of the flexibility of their supervisory frameworks and the typicality of their classification-based supervision approaches.

First, following Brexit, the UK amended its supervision laws for gene-editing technologies, enacting the *Genetic Technology (Precision Breeding) Act* in 2023. This legislation permits the use of techniques such as gene-editing to breed animals free from harmful diseases and authorizes the commercial application of gene-editing breeding technologies. Gene-edited organisms (including vertebrates) without exogenous fragments should be removed from the category of GMOs, but it is clearly required to establish a public registration system and take “animal welfare” as a prerequisite for commercialization. Compared to countries with strict supervision, the UK adopts a conditionally lenient policy: organisms produced through genome editing or other genetic technologies should not be supervised as GMOs if they could also be produced through conventional breeding methods.

Regarding food labeling, the *Genetic Technology (Precision Breeding) Act* [66] attaches great importance to the health and welfare of animals and their offspring. It mandates rigorous environmental impact assessments and comprehensive risk prevention procedures. It encourages and supports precision breeding technologies while safeguarding against potential risks to humans, animals, and the environment, embodying sustainable principles. In May 2025, the United Kingdom issued the *Genetic Technology (Precision Breeding) Act Regulation 2023*, though these provisions formally implement only the supervision clauses concerning precision-bred plants within the Act. This regulation focuses predominantly on the plant field and does not yet address technical specifications for precision breeding in animals.

In terms of animal welfare ethics, the standards for laboratory animal welfare in the UK are recognized globally as the strictest supervisory system. *The Animals (Scientific Procedures) Act* 1986 established three permits for conducting animal experiments: individual permits, project permits, and designated place permits. The animal welfare status supervisory system constitutes another vital measure concerning animal welfare in the UK. All experimental memorandums must undergo dual scrutiny by local ethical committees and inspectors, especially with laboratories subject to regular inspections. Furthermore, the Animal Perception Act, enacted in May 2021, is an important part of the action plan to improve animal welfare in the UK, both domestically and internationally, encompassing pets, livestock, and wildlife.

Second, the supervision of gene-edited animals in Australia focuses on process oversight and operates as an exemption system. Specifically, gene-edited animals that do not introduce exogenous genes (such as solely undergoing CRISPR knockout of endogenous genes) are exempt from approval and treated as natural mutations. Conversely, animals utilizing templates to introduce exogenous genes are subject to strict supervision as GMOs. This effectively exempts gene-edited animals without exogenous genes from the strict supervision applied to GMOs. The Food Standards Australia New Zealand (FSANZ) amended relevant definitions in the revised *Australia New Zealand Food Standards Code* published on 2 September 2025, aiming to promote innovation while safeguarding food safety.

These amendments include: (1) The definition of “GM food” was changed to “food derived from organisms (or cells) that have undergone genetic modification and contain exogenous DNA”. FSANZ deemed that this definition fails to cover emerging genetic technologies, potentially leading to over-supervision or gaps in coverage; (2) Naturally occurring or conventional breeding-induced gene changes are not considered genetic modification. The key basis is whether exogenous DNA is introduced; (3) Foods produced using emerging breeding techniques (such as genome editing) are not classified as GM if no exogenous DNA is introduced. GM foods still need to submit applications such as pre-market safety assessment to FSANZ and comply with the regulations on GM labels [67].

Regarding food labeling, gene-edited animal foods in Australia must be labeled as containing tissue from such animals. In terms of laboratory animal welfare and ethical review, Australia currently relies solely on a series of laws and regulations, like the *Best Research Methods for the Use of Animals in Scientific Research*, to safeguard the welfare of animals used in scientific experiments.

Third, the eclectic supervision in Thailand is a type of certification supervision. To advance the development of emerging biological breeding, Thailand’s Ministry of Agriculture issued the *Announcement on Applications for Certification of Gene-edited Organisms for Agricultural Use* on 11 July 2024, which allows certification of organisms developed through gene editing technology. This announcement aims to support the sustained development and commercialization of gene editing within agriculture, with broad applicability spanning animal, plant, and microbial domains. Additionally, Thailand issued the *Principles, Methods, and Conditions for the Certification of Plants Developed by Gene Editing Technology*, which stipulates the certification of gene-edited organisms. This document emphasizes safe agricultural applications and international cooperation to advance agricultural innovation and technological progress, highlighting the immense potential of this technology in improving agricultural biological varieties.

The Department of Fisheries promulgated the *Certification Standards and Procedures for Aquatic Animals Developed Using Gene Editing Technology* on 5 March 2025, regulating both certification and revocation processes. The scope of certification is explicitly limited to new aquatic animal varieties whose endogenous genes were modified through gene editing technology without introducing exogenous DNA. A mechanism is established to revoke certification should any of the following occur: the discovery of unintended trait expression in certified varieties, aquaculture environmental risks exceeding the standard, or false statements in submitted information for the declaration [68].

### 3.4. Appropriate Choice of Supervision Model for Gene-Edited Animals in China

Currently, China has not established separate regulations specifically for gene-edited animals and their food products; it still uses a relatively strict supervisory system for GM organisms (GMOs), which also has the typical characteristics of strict supervision. Research units for gene-edited animals still conduct safety assessments and submit applications in accordance with the supervision requirements for GM animals [69]. However, based on the differing technical characteristics of whether “detection results contain exogenous gene fragments”, gene-edited animals should be subject to sui generis supervision distinct from GM animals. A gradual transition from a strict supervision model to an eclectic one is an appropriate choice for China, to reflect efficiency and accuracy of supervision.

In addition to the aforementioned theory of supply and demand equilibrium, China’s transitional choice of supervision model for gene-edited animals can be supported by a cost–benefit analysis commonly used in law and economics. Specifically, the expected benefits and costs of alternative institutional options are analyzed to inform the selection of the optimal institutional choice, based on the results of the game of interests. This method can be used to analyze whether the cost–benefit of implementing a legal system is proportional, and it can also be adapted for horizontal comparison of the cost–benefit of implementing different legal systems.

First, compare the costs paid by the three supervision models. The economic analysis of law should focus on transaction costs [70]. Transaction costs have important redistributive and intergenerational equity implications [71]. (1) The strict supervision model for gene-edited animals is essentially an extension of the supervision system for GM animals, requiring only minor adjustments to the existing legal framework and incurring lower legislative costs. However, the strict supervision model inevitably involves cumbersome procedures such as review, certification and licensing, and the cost of legal implementation is high. (2) The open supervision model has an open attitude towards the certification of gene-edited animals, food labeling, and related matters. It could avoid cumbersome industrialization procedures. However, the law has a lagging effect, and with the rapid development of science and technology, the law needs to be revised in a timely manner, and there is a large duplication of legislative costs. (3) The eclectic supervision model focuses on “special treatment for special problems”, providing a degree of flexibility and relaxation for gene-edited animals that meet the criteria. The cost of substantial amendments to the law and its implementation is relatively small.

Second, compare the benefits gained from the three supervision models. From the perspective of law and economics, the benefits obtained from operating the law include soundness of legal system, public recognition, and guidance for industrial development. As gene editing technology matures and public awareness deepens, its applications will gradually become more accurate and safer. Against this background, global supervision of gene editing technology will gradually become more open and internationalized. It is important to note that liberalization will still be cautious and within controlled limits. The evaluation of the safety of gene editing technology is an ongoing process of updating and improvement, and relevant supervisory policies will be adjusted accordingly. Coupled with the ethical risks posed by gene-edited animals themselves, the eclectic supervision model is better able to appropriately loosen restrictions on industrializing gene-edited animals, gradually guiding and promoting the industry’s benign development, while maintaining overall legal system coherence and aligning with current public awareness levels of gene editing technology.

## 4. Categorization Path to Sui Generis Supervision System for Gene-Edited Animals in China

To advance the industrialization of gene-edited animals, the development of the supervision system for gene-edited animals requires top-level design, with ethical review mechanisms as a prior procedure, and support from full-process traceability and product labeling system. This approach can balance technological innovation and research ethics, while effectively safeguarding national food security and biosafety.

### 4.1. Strengthening the Overarching Design for the Categorized Supervision of Gene-Edited Animals

The Third Plenary Session of the 20th Central Committee of the Communist Party of China made further arrangements for the development of the socialist rule of law system with Chinese characteristics, calling for “strengthening legislation in key areas, emerging fields and foreign-related domains” [72]. Gene-edited animals, as an emerging field and a key area for national biotechnology development, should align with this policy direction to accelerate legislation and establish a robust top-level design. In particular, in combination with its application in the agricultural sector, the agricultural and rural competent departments, as the main body of government supervision, should formulate scientifically sound and effective supervision measures to ensure both the safety and reliability of the technology and the full realization of its value [57].

Specialized legislation would help ensure that the intensity of supervision is commensurate with risk, thereby protecting public and environmental safety while minimizing supervising restrictions on the development of emerging biotechnologies. At the same time, the Ministry of Agriculture and Rural Development should act as the core leading department in the construction of the supervision system for gene-edited animals, coordinating the relevant legislation, standard-setting and implementation coordination, which can ensure the unity and authority of the system design. Regarding policy formulation for gene-edited animals, the Ministry of Agriculture and Rural Affairs in China has initiated the development of the *Guideline for Safety Assessment of Gene-Edited Animals for Agricultural Use*. While the opinion letter explicitly states that this guideline primarily targets gene-edited livestock and poultry without introduced exogenous genes, as well as gene-edited aquatic animals, they do not yet cover all animal species. Consequently, there is an urgent need to accelerate the enactment of specialized legislation for the supervision of gene-edited animals and to promptly promulgate targeted laws and regulations. This will ensure the integrity and consistency of the legal system for biotechnology supervision.

First, the categorization of supervisory subjects. Currently, China has already implemented preliminary supervision in the declaration field of agricultural gene-edited plants. In 2022, the Ministry of Agriculture and Rural Affairs promulgated the *Guideline for the Safety Assessment of Gene-Edited Plants for Agricultural Use (Trial)*, which clarifies the declaration procedures for gene-edited plants based on the principles of categorized supervision and case-by-case analysis. The *Detailed Rules for the Review of Gene-Edited Plants for Agricultural Use* in 2023 further refined the relevant safety assessment content. The legislative experience in the plant field can be applied to the formulation of the *Guideline for the Safety Assessment of Gene-Edited Animals for Agricultural Use*.

However, given that gene-edited animals are more ethical than gene-edited plants, and that the maturity of the relevant technologies also varies significantly, legislative supervision in the animal field should comprehensively consider the differences between the two and should not be blindly replicated. The principle of categorized supervision may be adopted to establish a categorized supervision idea for gene-edited animals. The categorization standard should be based on whether exogenous genes are detectable in the final product, particularly for gene-edited products considered substantially equivalent to conventionally bred ones. This would enable categorized supervision between gene-edited animals where exogenous genetic material is undetectable in the final product and those where it remains detectable.

Theoretically, categorized supervision of gene-edited animals is necessary. (1) The presence or absence of exogenous genes in animal products significantly determines their safety and level of societal acceptance. Given that each scenario involves different risks and varying degrees of ethical sensitivity, coupled with differences in the maturity of technologies applied across scenarios, they should be subject to differentiated supervision [73]. (2) The legislative supervision models in other nations also demonstrate the remarkable effectiveness of categorized supervision for gene-edited animals. For instance, the National Agency of Drug and Food Control (BPOM) of Indonesia issued its *Regulation on GM Food* on 18 November 2024, indicating that food produced via gene editing techniques may be GM food or non-GM food. The former requires food safety approval from BPOM, while the latter may be treated as conventional food [74].

Practically, the categorized supervision of gene-edited animals is feasible. According to research findings published by the Institute of Crop Science, Chinese Academy of Agricultural Sciences/National South Breeding Research Institute, scientists have developed the RUBY-CRISPR visual editing system. By combining this system with four novel “suicide switches” (TKC components), the RUBY-TKC2 series of carriers was constructed. Surveys of gene-edited lines targeting the early-spiking gene *SE5* and the seedling-stage albino gene *YSA* in rice revealed that in the contemporary (T0) generation, all plants exhibiting coloration successfully achieved gene editing. By the next generation (T1), these “suicide switches” demonstrated potent efficacy, effectively clearing GM components. The most effective combination (TKC2.1) achieved 100% freedom from GM components in offspring plants, while other combinations attained clearance rates of at least 96%. This technology, incorporating the visualizable RUBY reporter gene, efficiently generates gene-edited plants without transgenesis. It resolves the issue of GM escape inherent in the original TKC gene-editing techniques. It is applicable not only to rice but also provides a crucial tool for gene editing in other plant and animal species [75], further offering the potential for categorized supervision of gene-edited animal products.

Second, clarify the supervision principles. Specialized legislation governing gene-edited animals should adhere to the following fundamental principles.

(1) Balance the modesty and openness of the law. Legislation should maintain prudence yet avoid an excessively negative stance that could cause stagnation in related fields. (1) The basis for formulating special legislation on the supervision of gene-edited animals should be grounded in the scientific principles of gene editing technology field, avoiding unrealistic requirements inconsistent with the field; (2) The object of assessment should be on gene-edited products rather than the gene editing process itself; (3) The prescribed content of the special legislation for the supervision of gene-edited animals should be operationally feasible, aiming to simplify detection items and related approval procedures; (4) Such legislation should facilitate the healthy development of agricultural animal breeding technologies [76].
(2) Adhere to the national bio-safety philosophy to safeguard biodiversity and ecological equilibrium. Under the supervision of government authorities such as ecological and environmental departments, supervision of gene-edited endangered animals and plants should adopt a hybrid model combining product and process, establishing systems for registration, labeling, and accountability [77].
(3) Implement categorized supervision of gene-edited animals, classifying gene-edited animal products based on detectability criteria, and establishing supervision regimes and assessment standards with different focuses.

It is important to note that implementing the top-level design of the categorization of gene-edited animals involves several aspects, including legislative and administrative costs. For instance, the formulation of specialized normative documents such as the *Guideline for the Safety Assessment of Gene-Edited Animals for Agricultural Use* involves the input of legislative resources, expert argumentation, public solicitation of opinions and many other aspects, all of which constitute legislative costs. In addition, after the promulgation and implementation of the normative documents, the costs incurred by the relevant administrative departments in the performance of their functions also fall within the scope of legislative costs. Meanwhile, it also offers the anticipated benefits of strengthening the legal system and supporting industrial growth.

Additionally, considering the challenges faced by relevant technical standards in practical implementation, such as the variability in implementation capacity across different regions and the need to coordinate imports and exports with countries that adopt the record-keeping system or follow the principle of substantial equivalence, it is important to improve the feasibility of specialized legislation on the typological supervision of gene-edited animals. This can be achieved by harmonizing it with the existing supervision framework for GM technology, thereby facilitating a smooth transition from a strict supervision model to a more balanced approach.

(1) For research and development projects that have begun safety evaluations under the GM animals supervisory pathway, an update channel should be established to allow them to voluntarily choose to continue applying the original *Regulations on the Safety Management of Agricultural Genetically Modified Organisms* to complete the approval process, or to provide supplementary certification indicating they do not contain DNA of exogenous origin and other assessment data that should be recognized under the new system.
(2) At the standard-setting level, the Ministry of Agriculture and Rural Development should collaborate with market supervision, health, and other departments to establish mutual recognition of testing methods and results for “whether or not it contains exogenous DNA,” to prevent duplicate testing by enterprises or barriers to product access caused by inconsistent departmental standards.

### 4.2. Establish Prior Procedure for Animal Welfare Ethics Review

Establishing animal welfare ethics review procedures facilitates the development of a supervision system for gene-edited animals. This review must precede technological implementation. Its outcomes act as both a prerequisite and a safeguard for deploying gene-edited animal products. Thus, it functions as an institutional checkpoint for the industrialization of gene-edited animal foods. Laboratory animal ethics review constitutes an ethical assessment and oversight of laboratory animals, compelling researchers to refine experimental methods, procedures, and means to conduct animal experiments with the best results and the least damage. The *Guideline for Ethical Review of Laboratory Animal Welfare (GBT 35892-2018)* of China stipulates that where an ethics committee finds an experiment compliant with welfare ethics provisions after review, it shall approve the ethical review and issue a review report [78].

However, in practice, some researchers lack understanding of the relevant regulations governing the ethical review of laboratory animals and even fail to grasp the significance of such reviews. Furthermore, within medical student education systems, the class hours for animal ethics are also very limited, the animal welfare and health issues, particularly ethical review of laboratory animal welfare, have long been neglected [79]. Existing issues primarily encompass: no emphasis on training in laboratory animal-related knowledge; inadequate recognition of the importance of welfare ethical review; lack of understanding regarding the content of welfare ethical review; and inadequate implementation of the “3R” principle [80]. Against this backdrop, the handling of laboratory animals used for gene editing is highly likely to fall short of ethical requirements, with traditional post-event supervision failing to cover the unique ethical risks associated with gene-edited animals. Therefore, establishing mandatory prior procedures for the animal welfare ethics review process would ensure ethical assessment precedes technological application. At present, China does not have a sound legal basis for the ethical review of gene-edited animals. It is urgent to actively promote the legislative process of related work to make it coordinated with the existing guidelines for the review of laboratory animals.

Specifically, to ensure that animal welfare ethical reviews do not become mere formalities, a prior procedure for animal welfare ethics review with clearly defined responsibilities and strict standards must be established. This should encompass multiple contents, including the review body, criteria and procedure, safety assessments and punishment mechanisms. Its feasibility is shown by both the implementation cost and the system interface.

(1) In terms of cost, the institutionalized operation of the ethical review process for animal welfare involves the legislative expense of revising normative documents like the *Regulations on the Administration of Laboratory Animals*, including the allocation of legislative resources such as legislative research and expert argumentation. It also includes the operational costs of the institutions conducting the ethical review and the administrative expenses of the Ministry of Agriculture and Rural Affairs along with other key agencies overseeing the performance of the ethical review by each institution. However, prior procedure for animal welfare ethical review helps prevent the risk of unethical commercialized products from the start, which can block market entry and hinder the transformation of research and development results. It also lowers the costs of fault tolerance and opportunity costs, among other benefits. Furthermore, the prior procedure for animal welfare ethical review can also yield expected benefits such as enhancing the identity of animal protection groups, increasing the efficiency of scientific and technological outcomes, and helping promote the products of gene-edited animals, which is cost-effective.
(2) From a system connection perspective, the practical constraints still present in the prior procedures for animal welfare ethical review, including the process’s idleness caused by limited institutional review capacity, and the circumvention of sensitive experiments across regions due to differing review standards between regions, among other issues. Based on this, on the one hand, the new ethical review process for gene-edited animals should be aligned with the *Guideline for Ethical Review of Laboratory Animal Welfare* and other existing standards, such as the main review components, criteria, procedures, disciplinary mechanisms, environmental safety, and food safety assessments. Additionally, specific review requirements should be incorporated into the guidelines to prevent institutional conflicts, building on the existing framework. On the other hand, a mechanism for mutual recognition of review results should be established, requiring all parties to recognize the conclusions of competent bodies to reduce duplicative reviews.

First, regarding the review bodies, a multi-tiered collaborative review system should be established. At the national level, the Ministry of Agriculture and Rural Affairs under the State Council could be specifically responsible for conducting re-examinations and supervising major, emerging, and contentious projects to take the overall situation into account. At the institutional level, all universities and research institutes engaged in gene-edited animal research should be required to establish independent review committees. Concurrently, a mechanism for blind review and peer review among these committees should be implemented to ensure comprehensive and balanced review perspectives.

Second, regarding the review process and standards, researchers must submit a detailed “Risk and Ethical Assessment Report for Gene-Edited Animals” to their institution’s review committee before commencing any genetic manipulation experiments. This facilitates a multi-angle assessment by the review committee.

(1) Animal welfare and ethical risk assessment. Different risk assessment standards should be established for hereditary gene-edited animals and non-hereditary gene-edited animals. Hereditary gene editing requires further evaluation of its potential impacts after entering the ecological genetic system. Distinctions should also be made regarding the application of gene editing in laboratory animals, animals used in agriculture, and wild species. Currently, applications in wild species are not extensive. For agricultural animals, the focus should be placed on evaluating their safety and off-target risks, so as to avoid unpredictable adverse impacts on animal welfare ethics. For laboratory animals, the focus is on the experiments and technology itself, including assessing potential animal health problems caused by off-target effects, and evaluating how the modified target traits of laboratory animals may subsequently affect their natural behavior, physiological functions, and quality of life.
(2) Environmental safety assessment. This evaluation should, in principle, be completed synchronously by the R&D team during the project design phase and serve as a prerequisite for initiating the prior review. Its core components include assessing the likelihood of accidental escape of gene-edited animals during experimentation or breeding and evaluating the possible ecological consequences of the edited traits diffusing through reproduction within wild populations.
(3) Food safety assessment. For gene-edited animals with food production as the ultimate goal, strict food safety evaluations must be conducted. The assessment should adhere to the principle of “substantial equivalence”. Only when proven to be as safe as conventional products through food safety assessment, or after all risks have been clearly identified and control measures have been established, shall the product qualify for subsequent applications for commercial production.

Finally, regarding the supervision and punishment mechanism, clear penalties should be established for actions without review or in contravention of review decisions. These may include project termination, recovery of funds, and listing of relevant responsible persons on a scientific research integrity blacklist. Ensure that institutions perform their duties through institutional deterrence.

### 4.3. Establish the Traceability Mechanism for the Industrial Chain and the Mechanism for Food Labeling

Compared to gene-edited plants and microorganisms, the development of gene-edited animals and their products has lagged, leading to delayed biosafety testing of gene-edited animal products. Furthermore, gene-edited animals involve animal welfare ethics. The safety concerns surrounding their use as food have become increasingly prominent, inevitably raising questions about the degree of public acceptance. Consumers have the right to know and the right to choose, particularly for products whose safety lacks definitive guarantees and which have not gained broad public acceptance. Safeguarding the public’s right to know of could be achieved through establishing full traceability across the gene-edited animals industry chain, implementing labeling systems for gene-edited foods, and fostering public participation in supervision.

First, a full-chain traceability mechanism should be established for gene-edited animal industry. Full-chain traceability not only safeguards the public’s right to know but also effectively prevents the risk of gene-edited animals escaping into the environment, thereby alleviating public concerns and easing supervision challenges. Taking the EU as an example, its proposed full-process record-keeping encompasses the entire process from supply link to operation link, documenting all processes of rearing, supply, and experimentation. Furthermore, the EU is committed to establishing an Animal Welfare Committee to oversee all stages of the process, evaluate all relevant personnel, actively seek methods to reduce or replace animal suffering, and openly accept public scrutiny [81].

The implementation pathways for establishing a full traceability mechanism across the entire gene-edited animal industry chain are as follows: (1) A national traceability information platform for gene-edited animals and their products could be established, realizing the traceability of the entire process from research and development, testing, approval application, to processing. (2) The State Council could spearhead the establishment of an inter-ministerial joint conference system for the safety management of gene-edited animals in agriculture. (3) An independent scientific advisory and ethical review committee for gene-edited animals could be established by referring to the ethical review committees for laboratory animal welfare. This would realize coordinated interaction across multiple sectors, including agriculture, food, health, environment, and inspection and quarantine, clarify responsibilities, and improve the supervision system for gene-edited animals [82].

Second, a labeling system should be established for food derived from gene-edited animals. Specifically, such labeling or other appropriate measures shall be applied to primary foods of animal origin and foods produced or processed therefrom. Such labeling should indicate whether gene editing technology is used or specify which gene-edited components it contains, enabling consumers to identify and select. On the one hand, the food labeling system safeguards consumers’ right to know and choose. On the other hand, such special labeling, combined with market confusion over positive and negative labeling, may raise public doubts about whether there is still a significant difference from conventional breeding, thereby reducing consumers’ willingness to purchase. Therefore, implementing a labeling system for gene-edited animal foods requires clear regulations on labeling methods and content, prohibiting negative labels such as “this product is not derived from gene editing” to avoid ambiguity. As gene-edited animal foods granted market approval have undergone safety reviews and corresponding certification licenses by the relevant departments, their edible and environmental safety is assured to a certain extent. Therefore, the labeling of gene-edited animal food can be voluntary, primarily to satisfy consumer demand for identifying gene-edited products.

Third, improve social public participation and the oversight mechanism. When applying emerging biological breeding techniques, consideration should be given to the feasibility of governmental supervision and consumer acceptance. The academic community and professionals should disseminate reliable information regarding gene-editing technologies such as CRISPR to gain public trust. The utilization of scientific and orderly public opinion guidance and the consultation mechanism involving multiple subjects, such as the government, experts, and public, can help improve the governance structure and decision-making legitimacy of gene editing technology [83]. Among government departments, the competent authority of agriculture and rural affairs under the State Council should be the main supervisory body, responsible for supervising and coordinating the national work. It should work in coordination with the competent authorities, such as the Ministry of Ecology and Environment under the State Council, the Ministry of Natural Resources under the State Council, the National Intellectual Property Administration, and be responsible for the relevant work of gene editing technology supervision within their respective duties. Namely, the Ministry of Agriculture and Rural Affairs, as the primary entity overseeing the traceability and labeling system, is responsible for building the platform, coordinating between departments, and performing other tasks to ensure consistent and effective implementation.

Consequently, supervision of research teams and personnel must be strengthened. While supporting them in conducting relevant applied research under the premise of abiding by Chinese laws and regulations is necessary, measures should be taken to prevent experimental studies from triggering bio-safety incidents, ethical incidents, or significant public opinions. This will avoid bringing about negative effects on research into emerging biotechnologies like gene-edited animals in China [84]. Regularly organizing seminars involving scientists, ethicists, legal experts, and others, alongside public hearings for relevant product applications, enables these forums to provide scientific guidance for public discussion while also furnishing scientific evidence for governmental decision-making.

It should be noted that the implementation costs of the full-chain traceability and food labeling system for gene-edited animals are relatively high. These costs mainly include the construction, operation, and maintenance of the traceability information platform, the compliance expenses for enterprises to achieve complete traceability and related labeling, and the law enforcement costs for market regulatory authorities to conduct sampling inspections of products entering the market. However, if the full-chain traceability and food labeling system can be implemented, the expected benefits it brings far outweigh the costs. Not only the essential supporting materials provided for ethical review and safety assessment during the experimental phase, but also food safety and environmental safety ensured during the industrialization stage, while reducing the risk of animal “escape” and genetic “drift”. Consistent and clear food labeling would better address the current issues of consumer misunderstanding of gene editing technology and confusion over negative labeling in the marketplace.

Additionally, to further improve the feasibility of implementing full-chain traceability and food labeling systems, they should be aligned with existing food labeling regulations.

(1) Regarding the methods of labeling, the labeling of gene-edited food should be separate from that of GM food, and “gene edited xx + description of the intended purpose of gene editing technology” (e.g., “gene edited tomatoes to increase yield and alleviate food problems”) should be clearly adopted to prevent consumer confusion.
(2) In the allocation of responsibilities within supervisory agencies, agricultural and rural authorities handle product certification and data entry before products reach the market, while the market supervision department manages labeling and investigates violations during circulation after products enter the market. The two departments can establish an information-sharing system to quickly initiate anti-unfair competition investigations into misleading advertising, such as “non-gene-edited food,” and to ensure consistent enforcement of food labeling rules.

## 5. Conclusions

The emergence of gene-edited animal research achievements presents opportunities for agricultural modernization and biological medicine advancement, whilst simultaneously posing technological risks, ethical risks, and supervision challenges. Establishing a scientific and reasonable sui generis supervision system for gene-edited animals requires grounding in basic national conditions of China, incorporating beneficial international experience, implementing categorized supervision and precise policy, to balance technological innovation with risk prevention and control. (1) A sound and comprehensive supervision framework must be grounded in robust top-level design. Specialized normative documents should be expeditiously issued to achieve the transition from a strict supervisory model to an eclectic supervisory model. Gene-edited animals and their products should be categorized based on whether exogenous genes can be detected in the final product, in order to respond to the institutional demands for the special and differentiated supervision of gene-edited animals and solve the problem of supervisory system deficiencies. (2) Animal welfare ethical review should be established as a necessary prior procedure, closely integrated with environmental safety and food safety assessment procedures to form a unified review and evaluation chain for gene-edited animals, which addresses the pronounced ethical risks, ensures animal welfare and biosafety, resolves bottlenecks in industrialization. (3) A full-process traceability mechanism should be established across the entire industrial chain. A voluntary labeling system for gene-edited foods should be established. Effectively ensure the safety of the entire process of research and development and promotion of gene-edited animals. In response to the public’s social concerns about gene-edited animal products and to address the issue of insufficient collaboration among the main bodies.

Compared to existing research, the innovations of this paper primarily encompassed three aspects: First, it systematically demonstrates the particularity of gene-edited animals relative to GM animals and gene-edited plants, arguing that existing frameworks should not be applied simply. It explicitly asserts the necessity and feasibility of constructing a sui generis supervision system for gene-edited animals. Second, it innovatively proposes a categorized supervision model for gene-edited animals, centered on the criterion of “whether exogenous genes can be detected in the final product” while accounting for application scenarios. This provides an operational institutional core for precise and differentiated supervision. Third, it proposes the prior review procedures for animal welfare ethics, the full-process traceability, and the resource utilization of food labels. These three elements are integrated as key procedural pillars to ensure the effective implementation of the sui generis supervision system for gene-edited animals, forming a complete supervision chain where substantive and procedural aspects complement each other.

Given the limitations of this study, future research can be improved in the following aspects:

First, this paper focuses on top-level design and normative construction of supervision systems. The proposed institutional recommendations, including categorized standards and review procedures, are mainly based on theoretical research. The administrative costs, technical feasibility, and specific impacts on industrial development during practical implementation remain to be further validated and refined through the collection of industry data, field research, and case studies. Empirical research should be further advanced in the future.

Second, this paper primarily addresses legislative and administrative oversight perspectives, whereas an efficient supervision system relies on multi-stakeholder coordination. Future research should further explore collaborative governance models involving governments, enterprises, research institutions, industry associations, and the social public, clarifying the roles and responsibilities of each entity in standard-setting, risk assessment, and supervision feedback.

Third, gene editing technology and its application scenarios are undergoing rapid evolution. The supervision framework proposed herein cannot foresee or encompass all novel risks and ethical dilemmas that may emerge in the future. Consequently, subsequent research should monitor the latest technological trends and conduct forward-looking institutional studies to ensure the ongoing adaptability and effectiveness of supervision policies.

## Figures and Tables

**Table 1:** Comparison of gene-edited plants, gene-edited animals and GM animals.

Comparison Dimension	Gene-Edited Plants	Gene-Edited Animals	GM Animals
Main Editing Objects	Somatic cell, callus, germ cell	Somatic cell, germ cell (See below for details on hereditary gene editing and non-hereditary gene editing)	Somatic cell, embryonic stem cell, germ cell
Whether includes germ-line inheritance	Yes [25]	Yes [26]	Yes [27]
Whether contains exogenous DNA	No [28]	No [29]	Yes [30]
Key ethical concerns	Off-target risk [31]	Off-target risk, animal welfare, intergenerational ethics	Environmental safety, animal health and welfare, human health and food safety [32]
International mainstream supervision models	Two types of supervisory frameworks: process-based supervision and product-based supervision [33]	Three types of supervisory models: open supervision, strict supervision, and eclectic supervision (See below for details on international comparisons)	Rigorous models dominate globally, primarily based on FDA standards and CAC standards [32]

## Data Availability

The data are available upon request from the corresponding author.
